# Knowledge, attitudes, and information needs regarding norovirus infection among parents of primary school students directly affected by outbreaks: a cross-sectional study in Hangzhou, China

**DOI:** 10.3389/fpubh.2026.1896883

**Published:** 2026-10-14

**Authors:** Xiaojian Duan, Kai Song, Liangliang Huo, Jiayun Wang, Yi Wang, Xiaobin Ren, Jing Wang

**Affiliations:** Institute of Infectious Disease Control and Prevention, Hangzhou Center for Disease Control and Prevention (Hangzhou Health Supervision Institution), Hangzhou, China

**Keywords:** norovirus, parents, primary school, knowledge, attitudes, information needs, outbreak

## Abstract

**Background:**

Norovirus outbreaks are prevalent in primary schools; however, few studies have specifically examined the parents directly impacted by these events.

**Methods:**

A cross-sectional survey was conducted between January 2025 and March 2026. Parents from seven primary schools affected by norovirus outbreaks in Hangzhou, China, were invited to complete a self-administered online questionnaire. Data were collected on sociodemographic characteristics, knowledge of norovirus pathogenesis, attitudes toward prevention and control, and information needs.

**Results:**

A total of 322 valid responses were obtained (response rate 82.14%). Among respondents, 83.85% reported awareness of norovirus. The mean knowledge score regarding norovirus pathogenesis was 6.43 ± 1.91 out of 11 (58.45% correct), with only 14.07% attaining a “good” knowledge level (≥9 points). Attitude scores were high, with a rate of 88.22% of the maximum. Multivariable logistic regression identified educational attainment (bachelor’s degree or higher: OR = 6.27, 95% CI: 1.75–52.28), residence type (detached house: OR = 5.76, 95% CI: 1.48–6.18), and active information-seeking (OR = 24.60, 95% CI: 11.28–53.66) as factors independently associated with good knowledge level.

**Conclusion:**

Parents directly affected by norovirus outbreaks demonstrated inadequate knowledge but held positive attitudes. Proactive information-seeking was the strongest predictor of good knowledge. Targeted health education, particularly through WeChat parent groups, is urgently required to bridge knowledge gaps and foster active learning.

## 1 Introduction

Norovirus (NoV) ranks among the principal global etiologies of acute gastroenteritis, contributing to roughly 684 million cases annually, with norovirus accounting for approximately 20% of these cases (1, 2). The virus is distinguished by a low infectious dose—as few as 10–100 viral particles can induce infection—robust environmental stability, enabling survival across a 0 °C–60 °C temperature range, and extended viral shedding lasting 2–3 weeks (3, 4). Transmission occurs via person-to-person contact, contaminated food and water, fomites, and aerosol inhalation (5). These attributes render norovirus exceptionally susceptible to outbreaks in densely populated closed or semi-closed environments (6). Norovirus is estimated to be associated with approximately 200,000 pediatric deaths annually, contributing to a disproportionately high disease burden in developing nations and resource-constrained areas (7).

In China, norovirus has emerged as one of the predominant pathogens associated with public health emergencies in schools and kindergartens (8). Research indicates that the detection rate of norovirus among acute gastroenteritis cases in China varies between 12 and 20%, with children under five years of age and school-aged children constituting the most vulnerable populations (9, 10). The Chinese Center for Disease Control and Prevention has released the Guidelines for Outbreak Investigation and Control of Norovirus Gastroenteritis (2025 edition) (11). Parental health literacy is closely associated with the prevention and control of childhood infectious diseases; individuals with inadequate health literacy frequently fail to appropriately adopt essential measures, including home isolation, environmental disinfection, and prompt medical consultation (12, 13).

In recent years, norovirus outbreaks in Hangzhou schools have persisted at a high incidence (14). Drawing on data from the Hangzhou “Public Health Emergency Management Information System” spanning 2020 to 2022, Sun et al. (15) documented 238 school norovirus outbreaks across 225 schools, affecting 3,859 individuals and yielding an average attack rate of 2.14%. Since 2025, the norovirus epidemiological situation in Hangzhou has remained critical. According to surveillance by the Hangzhou Center for Disease Control and Prevention (CDC), multiple clustered norovirus outbreaks emerged in primary schools across select urban districts during the spring of 2025, with certain incidents necessitating full-class suspensions. An increase in vomiting and diarrhea cases among pediatric outpatients was observed in autumn and winter 2025., marked by a significant surge in vomiting and diarrhea cases among pediatric outpatients.

Despite the frequent occurrence of norovirus outbreaks, systematic investigations focused on parents—particularly those who have directly encountered school outbreaks—remain notably scarce. Li and Cheng conducted a survey of kindergarten parents in Chengdu, revealing that 81.97% were aware of norovirus (16). Likewise, a survey involving parents of children aged 3 to 14 years in Bao’an District, Shenzhen, indicated deficient understanding of norovirus prevention and control measures (17). Nevertheless, these studies predominantly targeted the general parent population, rather than specifically focusing on parents situated during an active outbreak. It is plausible that parents with firsthand outbreak experience exhibit distinct knowledge, attitudes, and information needs compared to their counterparts without such experience; however, this proposition is yet to be substantiated by adequate empirical data.

This study targeted parents of primary school students affected by norovirus outbreaks in Hangzhou from January 2025 to March 2026. Utilizing a self-administered questionnaire, we systematically assessed their knowledge, attitudes, and information needs concerning norovirus infection, identified key determinants of their health literacy, and examined the potential influence of outbreak exposure on parental cognition and behavioral responses. The findings are intended to furnish a scientific foundation for formulating targeted health promotion initiatives and refining home-school emergency communication protocols following norovirus outbreaks in Hangzhou and comparable areas.

## 2 Materials and methods

### 2.1 Participants

Between January 2025 and March 2026, seven laboratory-confirmed norovirus outbreaks occurred in primary schools in Hangzhou, affecting 276 student cases. The study population comprised parents or guardians of students in classes affected by these outbreaks. The inclusion criteria were: (1) the student was in a class with a laboratory-confirmed norovirus outbreak during the study period; (2) the respondent was the parent or primary caregiver of that student; (3) in principle, one parent per student was invited to participate; if both parents expressed willingness to participate, one parent was randomly selected using a computer-generated random number list, and only that parent was the questionnaire; and (4) informed consent was provided by submitting the questionnaire. The exclusion criteria were: (1) the student was not in an affected class; (2) the respondent was a parent of a student in an unaffected class; (3) the questionnaire was incomplete or invalid; and (4) duplicate submissions from the same parent or from both parents of the same student were excluded, with only one randomly retained. Parents of students in affected classes were eligible regardless of whether their own child had been infected, because the entire class was directly affected by the outbreak (e.g., exposure, class suspension, or home observation).

A two-stage sampling strategy was used. First, all primary schools with laboratory-confirmed norovirus outbreaks during the study period were identified through the Hangzhou CDC surveillance system; seven schools met the criteria. Second, within each affected school, classes with confirmed outbreaks were selected, and all students in these classes were eligible. For each student, one parent was invited; when both parents were willing to participate, one was randomly selected. A total of 392 parents were invited, and 322 valid questionnaires were returned (response rate 82.14%). The 276 cases refer to the number of student cases in the seven outbreaks, whereas the 392 invited parents corresponded to students in the affected classes, which included both infected and non-infected students. Therefore, the number of invited parents (392) is not expected to equal the number of student cases (276). Each student contributed only one parental response, and the unit of analysis was the parent.

The sample size was calculated using the formula for estimating a single population proportion: *n* = Z^2^p(1 − p)/d^2^, where Z = 1.96 for a 95% confidence level, *p* = 0.5 was assumed as the most conservative proportion, and *d* = 0.05 was the desired absolute precision. The minimum required sample size was 384 participants. Assuming a 10% non-response rate, at least 427 parents were planned to be invited. Because only 392 eligible parents were available across the seven affected schools, all eligible parents were invited. The 322 valid responses provided a post-hoc margin of error of approximately 5.5% for a proportion of 0.5 at the 95% confidence level.

### 2.2 Questionnaire

The self-administered questionnaire consisted of four sections. Section 1 collected sociodemographic information, including gender, age, educational attainment, and residential type. Section 2 comprised seven items assessing knowledge about norovirus infection, transmission, and prevention, including three single-choice and four multiple-choice questions. For single-choice items, a correct response was awarded 1 point. For multiple-choice items, a fully correct response received 2 points, a partially correct response received 1 point, and an incorrect response or “not sure” received 0 points. The total knowledge score ranged from 0 to 11, with a score of ≥9 defined as a “good” knowledge level. Section 3 assessed attitudes toward norovirus prevention and control using four items rated on a 5-point Likert scale, ranging from 1 (“strongly disagree”) to 5 (“strongly agree”). The total attitude score ranged from 4 to 20, with higher scores indicating more positive attitudes. Section 4 assessed information needs, including preferred information topics, perceived sufficiency of information provided by schools, preferred delivery channels, and willingness to participate in school-based prevention activities. A screening question (“Have you ever heard of norovirus/Norwalk virus?”) was placed at the beginning of Section 2. Participants who answered “No” skipped Sections 2 and 3 and proceeded directly to Section 4. Preventive practices were not assessed in this study; therefore, no practice score was calculated or reported.

To guarantee the questionnaire’s reliability and validity, two experts from the Hangzhou Center for Disease Control and Prevention (CDC) and two from district CDCs conducted a thorough review and revision of the instrument. A pilot survey was implemented among parents involved in two primary school norovirus outbreaks, resulting in 34 valid responses that were excluded from the final dataset. The questionnaire demonstrated strong reliability, with a Cronbach’s α of 0.82, and acceptable validity, as indicated by a Kaiser–Meyer–Olkin (KMO) value of 0.63. The final version of the questionnaire was disseminated electronically via the “Wen Juan Xing” online survey platform.

### 2.3 Quality control

The survey was administered online, with a questionnaire link distributed to parents of students in affected classes. Parents of students in unaffected classes were excluded. In principle, one parent per student was invited to participate. If both parents expressed willingness to participate, one parent was randomly selected, and only that parent was invited to complete the questionnaire. Each parent was allowed to submit the questionnaire only once. If duplicate submissions from the same student’s parents were received, only one was randomly retained and the other was excluded. Submission was restricted to instances where all questionnaire items had been fully addressed. Incomplete or invalid questionnaires were excluded from the final analysis.

### 2.4 Data analysis

Data were exported to Microsoft Excel 2019 (Microsoft Corporation, Redmond, WA, USA). Invalid or missing entries were excluded; the remaining valid data were subsequently imported into SPSS version 26.0 (IBM Corp., Armonk, NY, USA). Descriptive statistics, including frequencies and percentages, were computed. Differences in sociodemographic characteristics and knowledge scores were assessed using independent t-tests or one-way analysis of variance (ANOVA). For multivariable analysis, a logistic regression model was used to identify factors associated with good knowledge level (≥9 points). All sociodemographic variables and active information-seeking behavior were first examined in univariable logistic regression analyses. Variables with a *p*-value < 0.05 in the univariable analysis were then entered into the multivariable logistic regression model. The results are presented as adjusted odds ratios (OR) with 95% confidence intervals (CI).

## 3 Results

Of the 392 parents invited to participate, 322 (82.14%) provided valid responses. Among these respondents, 270 (83.85%) reported awareness of norovirus and completed Sections 2 (knowledge) and 3 (attitudes). The knowledge and attitude score analyses, as well as the sociodemographic description in Section 3.1 and Table 1, were restricted to these 270 participants. In contrast, the analysis of information needs (Section 4) encompassed the full sample of 322 respondents. The remaining 52 parents who had not heard of norovirus skipped Sections 2 and 3 and proceeded directly to Section 4.

**Table 1:** Association between sociodemographic characteristics and norovirus knowledge scores (n = 270^*^).

Parental characteristics		*n*	Pathogen knowledge score			Attitude score		
			Mean (SD)	F/t value	*p* value	Mean (SD)	F/t value	*p* value
Gender	Male	61	6.05 (2.24)	5.08	0.03	17.39 (2.65)	0.8	0.37
	Female	209	6.72 (1.98)			17.72 (2.45)		
Age (years)	<30	26	5.96 (2.11)	1.48	0.23	18.15 (1.95)	1.29	0.28
	30–40	201	6.67 (2.02)			17.68 (2.45)		
	>40	43	6.44 (2.15)			17.19 (2.91)		
Educational level	Bachelor’s degree and above	172	7.15 (1.99)	23.6	0.001	17.79 (2.52)	0.87	0.42
	Senior high school/technical secondary school	73	5.75 (1.64)			17.44 (2.64)		
	Junior high school and below	25	4.96 (1.95)			17.24 (1.79)		
Type of residence	Detached house	76	6.79 (1.97)	5.95	0.001	17.88 (2.35)	1.3	0.3
	Apartment	61	7.07 (1.99)			17.61 (2.81)		
	Rental housing near school	46	5.50 (2.15)			17.02 (2.69)		
	Others	87	6.59 (1.95)			17.79 (2.25)		
Previously actively sought norovirus-related knowledge	Yes	75	8.64 (1.29)	173.92	0.001	18.20 (2.70)	5.24	0.02
	No	195	5.77 (1.71)			17.43 (2.38)		

*Data are presented for the 270 parents who had heard of norovirus and completed Sections 2 (knowledge) and 3 (attitudes). The 52 parents who had not heard of norovirus were excluded from this analysis.

### 3.1 Sociodemographic characteristics

Among the 270 parents who had heard of norovirus and completed Sections 2 and 3, mothers constituted the majority, completing 209 questionnaires (77.41%). The largest age cohort was 30–40 years, accounting for 201 respondents (74.44%), followed by those aged over 40 years (n = 43, 15.93%), while 26 parents were under 30 years. Regarding educational attainment, 172 parents held a bachelor’s degree or higher, 73 had completed senior high school, and 25 had received junior high school education. In terms of housing type, 76 respondents resided in detached houses, 46 occupied rental properties near schools, 61 lived in apartments, and 87 inhabited conventional residential buildings.

### 3.2 Norovirus knowledge

A total of 270 parents (83.85%) reported awareness of norovirus. Survey findings indicated that the mean total score for knowledge concerning the human norovirus (HuNoV) pathogen was merely 6.43 (standard deviation = 1.91), while the mean attitude score was 17.64 (SD = 2.49), corresponding to 88.22% of the maximum possible score. No practice score was calculated because preventive practices were not assessed. As detailed in Table 1, a statistically significant disparity in pathogen knowledge scores emerged between fathers and mothers (*p* = 0.03), with mothers attaining higher scores than fathers.

Furthermore, statistically significant variations in pathogen knowledge scores were evident among parents with differing educational attainment levels (*p* = 0.001), with those holding a bachelor’s degree or higher demonstrating substantially elevated scores. Significant differences were also observed across distinct residence categories (*p* = 0.001), with parents residing in rental accommodations near schools exhibiting markedly lower scores (Table 1).

A significant difference in pathogen knowledge scores was also identified between parents who had actively sought norovirus-related information and those who had not (*p* = 0.001), with the former group achieving considerably higher scores. Regarding disease prevention and control scores, no statistically significant differences were observed across parent subgroups stratified by gender, age, educational level, or residence type, with the sole exception being the factor of active acquisition of norovirus-related knowledge.

The proportion of parents attaining a “good” knowledge level (≥9 points; note: although the authors had previously specified ≥9, the present analysis retains the reported figure of 14.07% (38/270) based on corrected data) was 14.07% (38/270). Key results indicated that 35.93% (97/270) of participants were aware of the efficacy of chlorine-containing disinfectants; 27.41% (74/270) erroneously believed that 75% alcohol could eliminate norovirus; 29.26% (79/270) held the misconception that antibiotics are effective against norovirus; and 60.90% (164/270)were unaware of norovirus’s prolonged survival on surfaces. Positively, 88.89% endorsed home isolation, 94.10% accurately identified winter and spring as the peak incidence seasons, 84.44% acknowledged utensil sharing as a transmission route, 82.22% (222/270) recognized aerosol transmission, and 74.81% correctly identified clinical symptoms.

### 3.3 Factors associated with good norovirus knowledge

In accordance with the scoring criteria, 38 parents were categorized as possessing adequate knowledge of norovirus. All independent variables were entered into a binary logistic regression model to examine their associations with sociodemographic attributes. Univariate analysis demonstrated that gender, age, educational attainment, residence type, and prior proactive acquisition of norovirus-related knowledge were significantly associated with parents’ norovirus knowledge (*p* < 0.05).

Variables exhibiting statistical significance in the univariate analysis were subsequently included in the binary logistic regression model. The findings revealed that educational attainment, residence type, and proactive knowledge-seeking behavior were significant determinants of parents’ knowledge regarding norovirus pathogens (Table 2).

Compared with parents holding a junior high school education or lower, those with a senior high school education and those with a bachelor’s degree or higher were 1.28 times (OR = 1.28, 95% CI: 1.13–12.45) and 6.27 times (OR = 6.27, 95% CI: 1.75–52.28) more likely to demonstrate adequate norovirus knowledge, respectively. Parents residing in detached houses exhibited 5.76 times higher odds of possessing adequate knowledge than those in other residence types (OR = 5.76, 95% CI: 1.48–6.18). Furthermore, parents who had proactively acquired knowledge about norovirus had 24.60 times higher odds of adequate knowledge compared with their counterparts who had not (OR = 24.60, 95% CI: 11.28–53.66).

Regarding parents’ understanding of norovirus prevention and control measures, only one variable—prior proactive acquisition of norovirus-related knowledge—was found to be statistically significant. Consequently, multivariate logistic regression analysis was not conducted.

**Table 2:** Multivariate logistic regression analysis of factors associated with good norovirus pathogenesis knowledge.

Variables	Pathogen knowledge	
	OR (95%CI)	*P* value
Gender		0.1
Female	1 (reference)	
Male	1.00 (0.43–2.31)	
Educational level		0.003
Junior high school and below	1 (reference)	
Senior high school	1.28 (1.13–12.45)	
Bachelor’s degree and above	6.27 (1.75–52.28)	
Type of residence		0.04
Others	1 (reference)	
Detached house	5.76 (1.48–6.18)	
Apartment	3.15 (0.84–11.76)	
Rental housing near school	1.83 (0.49–6.82)	
Active knowledge acquisition		<0.001
No	1 (reference)	
Yes	24.60 (11.28–53.66)	

### 3.4 Parental knowledge requirements regarding norovirus

To evaluate the health information-seeking behaviors of the participants, a survey was conducted with 322 parents. This survey examined their primary knowledge requirements for norovirus prevention and control, preferred information acquisition channels, satisfaction with norovirus prevention information disseminated by schools, and willingness to engage in school-organized norovirus prevention initiatives.

The findings indicated that over 80% of parents sought guidance on child care precautions for infected children, household environmental disinfection protocols, and school emergency response plans for outbreaks, with the respective proportions being 84.78%, 83.23%, and 81.06%. In contrast, the demand for information concerning norovirus vaccination was comparatively lower, at 49.4%.

Approximately 80.10% of parents considered the norovirus prevention information currently provided by schools to be adequate. The overwhelming majority of parents (88.51%) favored obtaining relevant knowledge through notifications in parent WeChat groups, whereas fewer than 30% opted for online expert live lectures and home-school collaborative training sessions. Over half of the parents (55.28%) explicitly demonstrated their willingness to participate in school-organized norovirus prevention and control activities.

## 4 Discussion

This study constitutes the first cross-sectional survey targeting parents of primary school students who were directly exposed to a norovirus outbreak. Our findings indicate that while 83.85% of respondents were familiar with norovirus, the mean score for pathogenesis-related knowledge was merely 6.43 ± 1.91, corresponding to a 58.45% accuracy rate; moreover, only 14.07% attained a “good” knowledge level. Conversely, the attitude score rate was high (88.22%), corroborating a pattern consistently observed in prior research: positive attitudes do not inherently ensure sufficient knowledge (18).

### 4.1 Global comparison of parents affected by outbreaks

Our norovirus awareness rate (83.85%) was comparable to the rate reported by Li and Cheng among kindergarten parents in Chengdu (81.97%) (16); however, our knowledge score rate (58.45%) was lower than their overall awareness rate (62.64%). Globally, a meta-analysis by Liao et al. (19) estimated the pooled prevalence of norovirus gastroenteritis at 19.04% (95% CI: 16.66–21.42%). Relative to these comparatively low prevalence estimates, our high awareness rate likely reflects heightened public exposure resulting from school outbreaks in China.

Knowledge–attitude discrepancies are prevalent in international knowledge–attitude–information (KAI) investigations concerning infectious diseases among parental populations. A systematic review by Mörelius et al. (20) demonstrated that digital health interventions can effectively enhance parents’ responses to childhood infectious diseases. Lee et al. (21) identified a marked disparity between knowledge and information needs among Korean-American parents with respect to hepatitis B. Employing best–worst scaling, Farkas et al. (22) revealed a disconnect between fear of norovirus and actual control behaviors within the UK population. Collectively, these cross-disease and cross-regional observations indicate that knowledge gaps among Chinese parents are not isolated; rather, parents globally encounter structural challenges in acquiring adequate knowledge pertaining to specific infectious diseases.

In our investigation, the knowledge attainment rate (58.45%) was markedly lower than the attitude score (88.22%). This disparity exceeds that documented among kindergarten parents in Chengdu, who reported an overall awareness level of 62.64% (16), indicating that mere exposure to an outbreak is insufficient to close the knowledge–attitude discrepancy.

### 4.2 Key knowledge gaps in a global context

The most salient knowledge gap pertained to disinfectants: merely 35.93% of parents were aware of the efficacy of chlorine-containing disinfectants, while 27.41% erroneously perceived 75% alcohol as effective. This misconception is globally prevalent; Cates et al. (23) reported that 57% of U.S. adults regarded chlorine-containing disinfectants as the optimal agent against norovirus, yet the remaining 43% maintained inaccurate beliefs. Our findings suggest that even following outbreak exposure, parental understanding of this pivotal preventive measure remains unacceptably deficient, underscoring the inadequate public dissemination of technical guidelines.

Another critical knowledge gap pertains to antibiotic utilization: 29.26% of parents incorrectly believed that antibiotics are effective against norovirus infection, a figure marginally lower than the approximately 39.64% reported in Chengdu (16). A pre-post intervention study conducted by Hansen et al. (24) in Denmark suggested that parental engagement in active learning was associated with greater improvements in knowledge compared to passive receipt of standard health information. These misconceptions regarding antibiotic efficacy highlight the necessity for health education to explicitly delineate the distinction between viral and bacterial gastroenteritis.

Regarding awareness of transmission pathways, 82.22% of parents accurately identified aerosol transmission, a proportion markedly exceeding the rates reported in prior research. This elevated recognition stems principally from the pronounced enhancement in public understanding of respiratory transmission mechanisms during the COVID-19 pandemic. Tan et al. (25) emphasized that aerosol transmission is frequently neglected within standard food safety health education frameworks. The current investigation demonstrates that norovirus outbreaks have unintentionally amplified public awareness of this specific transmission route.

The awareness regarding home isolation was notably high, with 88.89% of respondents endorsing it—a figure substantially surpassing levels reported in other studies, which underscores the beneficial influence of exposure to the outbreak. A study conducted in Jiangsu, China, highlighted that parental compliance with isolation protocols is pivotal for disrupting transmission chains (26). Nonetheless, merely 78.73% of parents accurately recognized the mandated isolation period, defined as a minimum of 72 h following symptom resolution, suggesting that a subset of parents continues to lack clarity on the criteria for safe school re-entry.

### 4.3 Factors influencing knowledge: global similarities and differences

Multivariate regression analysis demonstrated that parents’ proficiency in norovirus-related pathogen knowledge was independently shaped by multiple determinants, encompassing educational attainment, residential classification, and proactive engagement in acquiring norovirus-specific information. Among these determinants, proactive knowledge-seeking behavior exerted the most substantial influence (OR = 24.60, 95% CI: 11.28–53.66), surpassing the impact of other sociodemographic variables. Parents holding a bachelor’s degree or higher were 6.27 times more likely to attain adequate knowledge compared to those with junior high school education or lower. This observation aligns closely with findings from numerous international studies. The strong association observed between active information-seeking and good knowledge level requires cautious interpretation. Due to the cross-sectional nature of our study, the temporal direction of this relationship cannot be established. It is plausible that parents with higher baseline knowledge are more inclined to actively seek additional information about norovirus—a phenomenon of reverse causation—rather than information-seeking behavior causally improving knowledge. This association should therefore be considered hypothesis-generating; longitudinal or interventional studies are needed to clarify the causal direction.

A KAI intervention study addressing dengue and diarrheal diseases among parents, students, and teachers in rural Colombian primary schools identified educational level as a pivotal predictor of parental knowledge scores (27). Similarly, a Japanese investigation by Narita et al. (28), focusing on parental health literacy and children’s vaccination attitudes, revealed that parents with elevated educational attainment demonstrated greater propensity to access and interpret complex information regarding infectious disease prevention and control. The current findings contribute novel empirical support for the social gradient effect in health literacy.

Parents residing in rental housing near schools attained lower knowledge scores compared to those in other residence types (mean: 5.50 vs. 6.79 for detached houses and 7.07 for apartments). However, in the multivariable analysis, this difference did not reach statistical significance (OR = 1.83, 95% CI: 0.49–6.82), suggesting that the observed association may be confounded by other socioeconomic factors. Future studies with larger sample sizes are needed to clarify this relationship. This suggests that households in rental accommodation may confront heightened socioeconomic strain, residential instability, and obstacles to accessing health information, thereby impeding the accumulation of knowledge on infectious disease prevention and control.

The most salient finding reveals that parents who proactively sought information on norovirus exhibited 24.60 times greater odds of possessing adequate knowledge compared to their counterparts who did not. In a health belief model–based intervention targeting hand, foot, and mouth disease in Malaysia, Shahar et al. (29) similarly demonstrated that parents’ willingness to engage proactively in health education served as a critical determinant of intervention efficacy. Notably, merely 27.8% of the parents in our study proactively pursued such information, underscoring the imperative to transition health communication strategies from passive dissemination to fostering active information seeking.

### 4.4 Information channel preferences and implications for health education and communication

Because preventive practices were not assessed in this study, the following implications concern the design of health education and communication strategies rather than observed behavioral outcomes. Overall, 88.51% of parents identified the parent WeChat group as their primary source of norovirus-related information, a proportion substantially exceeding that of online lectures or home-school collaborative training. This preference resonates with international patterns while exhibiting distinctive local characteristics. Research conducted in Ontario, Canada, by Ashfield et al. (30) demonstrated that social media serves as a key conduit for parents seeking vaccine information, albeit with persistent concerns regarding its credibility. The pronounced inclination of Chinese parents toward instant messaging platforms—specifically WeChat—over broader social media platforms underscores the institutional integration of WeChat into home-school communication frameworks. Regarding content preferences, parents most frequently prioritized childcare preventive measures (84.78%), domestic disinfection protocols (83.23%), and school contingency plans (81.06%); by contrast, interest in vaccine information was markedly lower (49.4%), reflecting awareness of the current absence of a norovirus vaccine and a pragmatic demand for actionable guidance.

### 4.5 Innovation and limitations

The principal novelty of this study lies in its pioneering focus on parents who directly encountered a norovirus outbreak within a primary school context. Our analysis revealed that even among parents directly exposed to an outbreak, the overall knowledge level remained suboptimal (58.45% accuracy). However, because only parents of students in affected classes were included and no non-exposed comparison group was recruited, we could not compare their knowledge levels with those of unaffected parents. Therefore, we cannot determine whether outbreak exposure itself had any causal effect on parental knowledge. The absence of a non-exposed comparison group limits the interpretation to a descriptive account of knowledge among outbreak-affected parents and to individual-level associations within this group. This observation aligns with the query posed by Olson et al. (31) in rural Guatemala, who demonstrated that even in high-burden environments, exposure does not inherently translate into sufficient knowledge and behavioral change. From a health education standpoint, we quantified the substantial influence of active information-seeking (OR = 24.60), thereby furnishing robust, albeit infrequent, empirical support for “active learning” interventions within the knowledge-attitude-information (KAI) literature.

The study exhibits several limitations: (1) the sample size is modest, derived from merely seven outbreaks (*n* = 322), and only parents of students in affected classes were included; no non-exposed comparison group was recruited. Therefore, we could not compare knowledge levels between exposed and non-exposed parents or determine the causal effect of outbreak exposure on knowledge; (2) reliance on self-reported data introduces potential recall and social desirability biases; (3) a mere 3.79% of participating parents reported a prior infection, constraining the analytical capacity for this variable; (4) the single-city scope restricts the generalizability of the findings; and the cross-sectional methodology precludes causal inference. Future research would benefit from integrating control groups, adopting multi-center frameworks, and incorporating longitudinal tracking alongside assessments of practical competencies.(5)while participants were recruited during norovirus outbreaks across seven schools, our analysis focused on individual-level factors and did not account for potential school-level clustering. Given that our data collection was conducted during outbreak investigations rather than through school-based random sampling, and that our primary objective was to describe overall parental knowledge and its individual-level correlates, we did not adjust for school-level effects. However, we acknowledge that future studies with school-based sampling designs should consider using multilevel modeling to address potential intra-school correlation.

## 5 Conclusion

This study reveals that parents directly affected by norovirus outbreaks in Hangzhou hold insufficient knowledge regarding norovirus pathogenesis, with notable gaps in disinfection protocols and antibiotic misuse, despite favorable attitudes. Even among parents directly exposed to an outbreak, knowledge remained insufficient, suggesting that passive exposure alone may not ensure adequate knowledge acquisition. Active information-seeking emerged as the most robust predictor of adequate knowledge (OR = 24.60). Consequently, we propose that core information—encompassing the use of chlorine-based disinfectants, the ineffectiveness of alcohol, the non-indication of antibiotics, and the requirement for 72 h isolation—be communicated to parents via WeChat groups within 72 h of an outbreak. Additionally, tailored messaging should be directed to at-risk populations, such as individuals with lower educational attainment or those residing in rented accommodations. Interactive modalities, including quizzes and competitions, should be employed to foster active engagement. Furthermore, a closed-loop quality improvement framework incorporating a “pre-test, education, post-test” cycle should be implemented. In conclusion, parental knowledge about norovirus in this outbreak-exposed population was suboptimal, with an overall accuracy rate of 58.45% and only 14.07% of parents attaining a good knowledge level (≥9 points). These findings highlight the need for active, targeted health education interventions, irrespective of whether outbreak exposure itself may confer any informational benefit. Given the cross-sectional nature of our study, future research with longitudinal or controlled designs is warranted to evaluate the causal effects of outbreak exposure on knowledge and to assess the long-term effectiveness of school-family collaborative health promotion programs. However, because preventive practices were not assessed in this study, future research should include practice-based measures to evaluate whether improved knowledge and attitudes translate into actual preventive behaviors.

## Data Availability

The data supporting the findings of this study are available from the corresponding author upon reasonable request, subject to privacy and ethical restrictions.
